# Can Undergraduate Dental Education be Online and Virtual During the COVID-19 Era? Clinical Training as a Crucial Element of Practical Competencies

**DOI:** 10.15694/mep.2020.000215.1

**Published:** 2020-09-30

**Authors:** Rayan Sharka, Hassan Abed, Arkadiusz Dziedzic

**Affiliations:** 1 Faculty of Dentistry Oral & Craniofacial Sciences; 2 Medical University of Silesia

**Keywords:** e-learning, blended learning, virtual reality simulation, dental education, COVID-19

## Abstract

This article was migrated. The article was marked as recommended.

The COVID-19 pandemic has brought ample challenges to clinical dental education all over the world. Dental schools had to adopt diverse strategies as a result of the exceptional circumstances, to provide a safe environment for their students, faculties, and patients. Despite the broad implementation of innovative educational tools in the form of blended learning, virtual reality simulators (VRS), and virtual learning environment, dental students expressed their willingness to restore on-site practical lessons, developing their clinical skills with patients’ presence.

It is believed that undergraduate dental education (UDE) during post COVID-19 pandemic lock-down might require substantial organisational changes, adequate adjustments of dental curricula and novel educational approaches in order to maintain a high level of UDE. This should be delivered by utilising the blended teaching methods, with core involvement of traditional clinical sessions and safety preventative measures arranged by dental faculties, allowing a safe return to dental schools for at least essential clinical sessions.

This personal view aims to emphasis the need for re-establishment and continuity of crucial clinical and practical dental training during ‘new normal’ dental education era, as an integrated and unique element of UDE, which can be only partially substituted by online learning programs.

## Introduction

Covid-19 has brought ample challenges to education, hampering the global health system and economy, as well as society at large. Inevitably, both medical and dental schools instantly adopted various, diverse strategies for coping with the exceptional situation to provide a safe environment for their students, staff, and patients aligned to various sectors of high education and economies. As a result, governments introduced regulations concerning social distancing, movement restrictions, and nation lockdown. This led to the complete suspension of universities and faculties on-site educational activities, including dental schools, except for some dental school hospitals which provided urgent and emergency dental care ( Quinn *et al*., 2020).

Undergraduate dental students, clinical teachers, and dental educators were forced to stay at home, while their practical clinical sessions were substituted with other forms of dental education. Dental educators had to come up with new, innovative ways of ensuring the partial continuation of knowledge dissemination based mainly on using online platforms and other informative technology methods, including primarily live streaming and video conferencing lectures, with virtual problem-based tutorials among others adopted to deliver curricula programs and motivate students to continue learning ( Liu *et al*., 2020).

## Traditional clinical training as a crucial element of undergraduate dental education

Following the excessive use of online tools over the last few months and in discussion with our dental students (certainly using online communication), it has been concluded that there is no substitute for actual clinical cases involving patients in clinics. Most students clearly expressed their willingness to safely return to dental school for practical sessions as soon as possible. Moreover, clinical teachers and students accept that clinical-based education is highly specific and cannot be replaced with any, even the most advanced, technologies at present. Eventually, clinical teachers and students must carry on with their duties and live in the ‘new normal’.

Equally, robust and fully structured dental training in variable teaching/learning environments has been a core, fundamental element of UDE globally, regardless of local preferences. The face-to-face, trainer-student teaching pathway is still considered as the best educational practice and now, some dentists may regret the fact there was not enough practical training with direct patient involvement. Unfortunately, this lack of a ‘real scenario’ might have an immense impact on the future career choices of dental practitioners.

The outbreak of COVID-19 has raised the following questions: Is there any need for a substantial change in the UDE curricula as a result of COVID-19? Should the UDE curricula be dramatically altered or reduced? Is there genuine evidence-based data suggesting that online training can replace the traditional teaching methods in dental clinical subjects? Shall we be optimistic about the future of mainly online/virtual UDE?

## The role of blended learning and e-learning methods during COVID-19

As we endeavour to provide competent instruction in our curriculum during the current pandemic, it is crucial to reconsider the effective adoption and implementation of various e-learning and blended methods. Undoubtedly, due to the sudden high demand for the digitalised environment during COVID-19, e-learning has become mainstream mainly due to safety and epidemiological measures. Hence, it is important to highlight that e-learning is not only broadcast of synchronous and asynchronous educational information in an electronic format. The curriculum content should be revised to discuss the prospect of dental modules and courses that might be run entirely online or as a mixture of traditional class learning with online learning (blended learning). This method merges the advantages of e-learning, whereby teaching is without time and space restraints, with additional input from face-to-face teaching. Dental students’ satisfaction with the e-learning resources delivered in a blended approach was relatively good and they performed better academically ( Jeganathan and Fleming, 2020; Yashwant *et al.,* 2020).

However, from our experience when learning technologies are presented, consideration is usually paid to the implementation of technology, while there is insufficient time, effort or funds allocated to the creation of appropriate e-learning resources content to produce a prosperous blended program. Relying solely on online education seems to be justified and rational, as long as it provides theoretical information, case presentations, essential explanations, and the rationale for the next clinical stages, mainly for non-clinical teaching of the first three years of BDS/DDS programs. However, for clinical years, it would be challenging to adopt such a simplified and online-based long-term approach to replace on-site training, as well as purely clinical practice.

**Figure 1.**
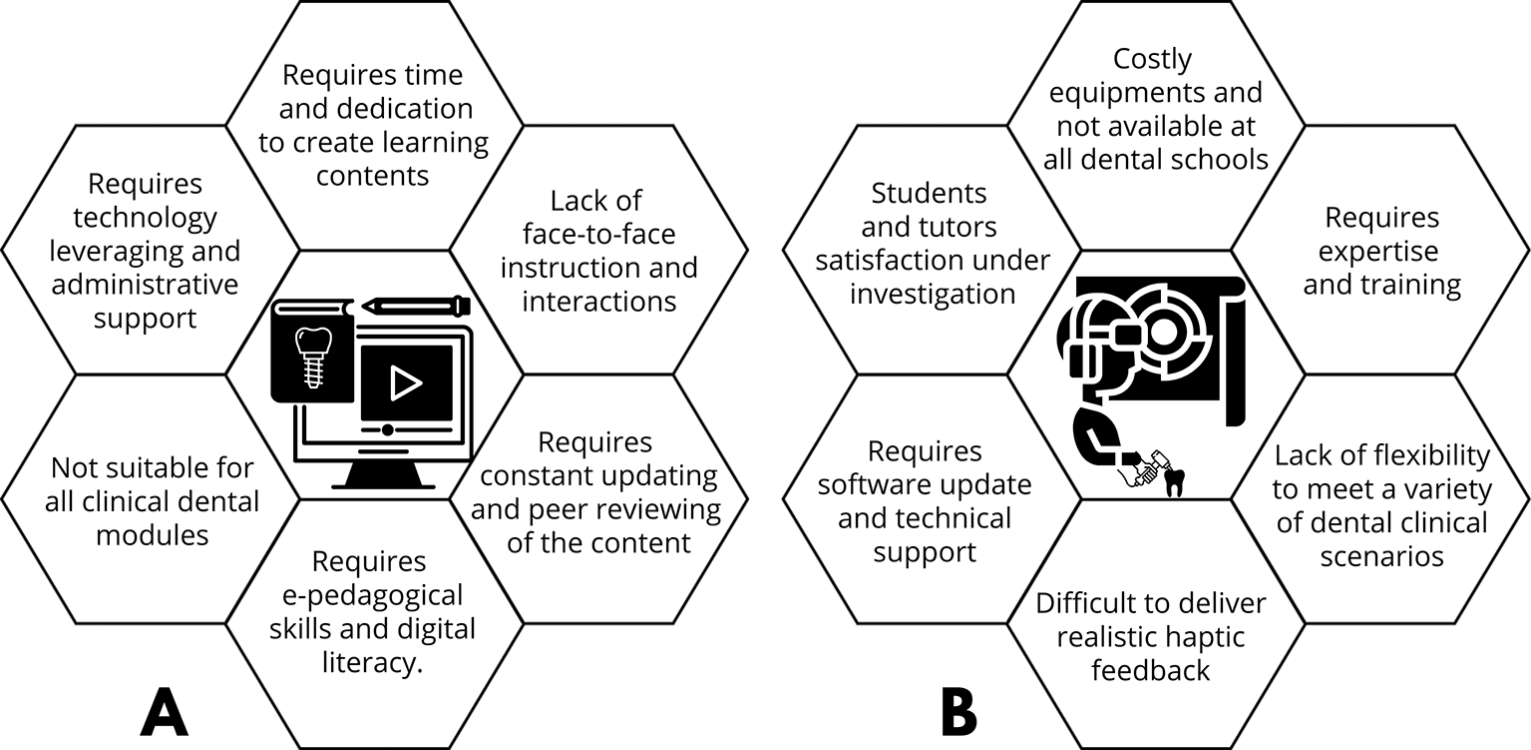
A & B: summarises some limitations of using e-learning and VRS in UDE.

For dental educators and clinical teachers, the difficulties with adopting e-learning are associated with their basic needs for the required technical skills together with a lack of confidence, which can frustrate their enthusiasm to engage with the development and offering of online learning. This seems to be associated with a lack of incentives and motivation to engage with online or e-learning before the pandemic. Also, the lack of dental school infrastructure as an ongoing challenge in dental education cannot be excluded ( Figure 1, A).

In addition, we would support the effective use of the Virtual Learning Environment (VLE) within dental schools, which provides alternative assistance for teaching and learning by supporting e-learning resources electronically and facilitating engagement in interactive characteristics, such as assessments, assignments, and discussions. Integrating such a learning environment for managing educational material, monitoring students and educators, and customising learning and teaching processes is required. Among the most popular dedicated VLE adopted in dental schools are Moodle and Blackboard that assist in advancing learning activities in a well-organised and prospective manner. Although some dental schools have implemented such platforms or created their VLE that is suitably built with their contexts of use, there is often an issue regarding the technical skills and accessible technology for dental educators and students. Hence, training and workshops to reduce the technical challenges and improve skills should be considered.

## Virtual Reality Simulation (VRS)

Although virtual reality simulation (VRS) for dental education training and the use of the most advanced technology showed promising results, it is no established educational standards for VRS or their associated exercises, as well as requiring substantial financial investment. For instance, ‘tactile sensation’ is a unique, individual human feature and even using VRS training simulators branded as mixing virtual reality with reality for virtual cavity and crown preparation, supported by state-of-the-art online learning technology platforms and associated technologies, it will be challenging to deliver a genuinely clinical scenario, although, the virtual clinical sessions can be designed, monitored, and repeated to expedite learning.

Without a doubt, dental simulation facilities have become an efficient way to teach undergraduate students’ safely while considerably improving their pre-clinical skills and reduce the required clinical teacher supervision time as well as promote a more learner-centred approach to the complex technical and clinical skills required to conduct clinical oral healthcare during a pandemic ( Murbay *et al.,* 2020; Liu *et al.,* 2020). However, there is no real patient interaction, an immensely important aspect of dental care and most importantly, very few dental schools have such VRS equipments, nor the in-house expertise for haptic technology to be implemented effectively and promptly ( Figure 1, B).

## Provision of a safe clinical teaching and learning environment in the COVID-19 Era

Rather than utilising a complete suspension approach or drastically reduced on-site and face-to-face training for dental students, especially for those in final years, dental schools administrators would consider resuming practical sessions of UDE following a scheduled time slot and a well-structured rotation system for clinical training sessions with substantial limitation of students’ number and contact would be one of the provisional plans. However, keeping enough time slots between student’s groups to allow generous ventilation and air replacement still a challenge.

Online training and workshop about infection control practices should be delivered. Strict cross-infection control measures in university teaching clinics, e.g. additional air purifiers, ozone generators, UV technology, HEPA filter extractors, would be considered on a broader scale to help re-establish the pre-COVID UDE, following risk assessment and compliance with national and government guidance and core standard operating procedures for the de-escalation phase ( Iyer *et al.,* 2020). Also, it would be suggested that the rapid molecular test COVID-19 screening of staff and students, and subsequent pre-appointment rapid testing for patients attending university teaching clinics will secure optimal and comprehensive UDE programs.

## Conclusion

The COVID-19 pandemic has brought critical hardships for clinical dentistry and educational institutions. As both teachers and clinicians, we must act sensibly and responsibly not to deter the young generation of dentists, offering a new technology that can substitute traditional dentistry teaching. While the traditional on-site training with patient involvement is still considered as a core element and works well, the UDE should be optimised and adjusted by combining traditional methods with new approaches. The creation of blended learning, replacing lectures with online tutorials seems to be in the right direction.

We would welcome decision-makers and stakeholders to take into consideration the long-term effect of deferred practical clinical sessions on the young generation of dental students. Overall, we as educators are responsible for the future high quality of dental care and comprehensive care provision for, especially vulnerable patients. The current COVID-19 pandemic should be regarded as an excellent opportunity for dental educators and the health care faculties at large to review their clinical practices and implement measures that will keep the future of the next generation of dentists alive.

## Take Home Messages

• Adopting online education seems to be justified and rational during COVID 19 era. However, for clinical education, it would be challenging to adopt such a simplified and online-based as a long-term approach to replace on-site training.
• Resuming clinical training sessions with substantial limitation of students’ number and additional measures to reduce the risks would be one of the provisional plans.
• Research is necessarily required to build evidence on the efficient use of technologies for clinical training for the long term.

## Notes On Contributors

**Rayan M. Sharka, BDS, MDSc,** is a clinical teaching assisstant and Lecturer in Prosthodontics, Departmentof Oral and Maxillofacial Surgery and Diagnostic Sciences, Faculty of Dentistry, Umm Al-Qura University. A PhD candidate, Centre for Dental Education, Faculty of Dentistry, Oral & Craniofacial Sciences, King’s College London. ORCID ID: https://orcid.org/0000-0002-9475-510X

**Hassan H. Abed, BDS, MSc,** is a special care dentist and a PhD candidate in Special Care Dentistry, Centre for Oral, Clinical & Translational Sciences, Faculty of Dentistry, Oral and Craniofacial Sciences King’s College London. ORCID ID: https://orcid.org/0000-0003-3817-3938

**Arkadiusz Dziedzic, DDS, MFDS/RCPS, PhD,** is an Assistant professor, Department of Conservative Dentistry with Endodontics, Medical University of Silesia. ORCID ID: https://orcid.org/0000-0003-0022-8382
