# Supplementary material for: Can Undergraduate Dental Education be Online and Virtual During the COVID-19 Era? Clinical Training as a Crucial Element of Practical Competencies
Source: MedEdPublish (2016). 2020 Sep 30;9:215. [Version 1] doi: 10.15694/mep.2020.000215.1 (PMC10699369; doi:10.15694/mep.2020.000215.1)
Supplement: Peer review history

# Reviewer report: Reviewer response for version 1

**DOI:** 10.21956/mep.20193.r31243

**Published:** 2020-10-18

**Authors:** Mushriq Abid

This review has been migrated. The reviewer awarded 5 stars out of 5 Interesting and very well organized paper that summarizes the challenges that teachers, students and clinicians face during the pandemic. Different fields including the educational sectors have been affected by Covid-19. For the time being online training is a good substitute but not the perfect one. I believe that virtual training cannot and will not substitute clinical or traditional training.

Reviewer Expertise:

NA

No decision status is available

# Reviewer report: Reviewer response for version 1

**DOI:** 10.21956/mep.20193.r31244

**Published:** 2020-10-14

**Authors:** Mohsen Aljabri

This review has been migrated. The reviewer awarded 5 stars out of 5 This is really an interesting paper. In fact, UG and even PG students will have several challenges due to the current pandimic situation. I am not sure what ’habtics in dentistry’ can add and help us during the clinical training? What are the benefits in general and specifically the cost benefits?

Reviewer Expertise:

NA

No decision status is available

# Reviewer report: Reviewer response for version 1

**DOI:** 10.21956/mep.20193.r31242

**Published:** 2020-10-14

**Authors:** Naif Ganadely

This review has been migrated. The reviewer awarded 5 stars out of 5 Virtual classes and virtual training can’t be a substitute for the traditional method. Students can be easily distracted at home. There are different solutions can be done to adopt with the new normal. For example: open the dental school 7 days a week and hire more faculty. I believe that VR can only be an additional method of learning. By the way, it is really interesting paper. Thank you.

Reviewer Expertise:

NA

No decision status is available

# Reviewer report: Reviewer response for version 1

**DOI:** 10.21956/mep.20193.r31241

**Published:** 2020-10-12

**Authors:** Mohamed Al-Eraky

**Affiliations:** 1 University of Dammam

This review has been migrated. The reviewer awarded 4 stars out of 5 This is an interesting article, yet the question in the title cannot be answered by a simple Yes or No. It all depends on the competencies we need in future graduates of dental schools and the expected roles in the society. Will they offer consultations online, or they have to practice certain skills on patients? Then it’s crucial to decide ‘when’, ‘why’ and ‘for how long’ we need our current dental students to come to practice in the medical schools and when to trust them to learn other things on their own or at a distance. We have a great responsibility to keep our standards high and safeguard the quality of our graduates, particularly in this era of uncertainty, which was perfectly advocated by the authors. Good luck!

Reviewer Expertise:

NA

No decision status is available
